# Comparison of Gas Chromatography-Mass Spectrometry and Gas Chromatography-Tandem Mass Spectrometry with Electron Ionization and Negative-Ion Chemical Ionization for Analyses of Pesticides at Trace Levels in Atmospheric Samples

**DOI:** 10.4137/aci.s1005

**Published:** 2008-09-09

**Authors:** Renata Raina, Patricia Hall

**Affiliations:** University of Regina, Department of Chemistry and Biochemistry, and Trace Analysis Facility (TAF), 3737 Wascana Parkway, Regina, SK, Canada S4S 0A2

**Keywords:** gas chromatography, negative ion chemical ionization, mass spectrometry, pesticide analysis, atmospheric samples

## Abstract

A comparison of detection limits of gas chromatography-mass spectrometry (GC-MS) in selected ion monitoring (SIM) with gas chromatography-tandem mass spectrometry (GC-MS/MS) in selected reaction monitoring (SRM) mode with both electron ionization (EI) and negative-ion chemical ionization (NCI) are presented for over 50 pesticides ranging from organochlorines (OCs), organophosphorus pesticides (OPs) and pre-emergent herbicides used in the Canadian prairies (triallate, trifluralin, ethalfluralin). The developed GC-EI/SIM, GC-NCI/SIM, and GC-NCI/SRM are suitable for the determination of pesticides in air sample extracts at concentrations <100 pg μL^−1^ (<100 pg m^−3^ in air). No one method could be used to analyze the range of pre-emergent herbicides, OPs, and OCs investigated. In general GC-NCI/SIM provided the lowest method detection limits (MDLs commonly 2.5–10 pg μL^−1^) along with best confirmation (<25% RSD of ion ratio), while GC-NCI/SRM is recommended for use where added selectivity or confirmation is required (such as parathion-ethyl, tokuthion, carbofenothion). GC-EI/SRM at concentration <100 pg μL^−1^ was not suitable for most pesticides. GC-EI/SIM was more prone to interference issues than NCI methods, but gave good sensitivity (MDLs 1–10 pg μL^−1^) for pesticides with poor NCI response (OPs: sulfotep, phorate, aspon, ethion, and OCs: alachlor, aldrin, perthane, and DDE, DDD, DDT).

## Introduction

Concerns over the inherent toxicity of pesticides and their potential as endocrine disruptors has lead to increased interest in studying the presence of pesticides in environmental media including atmospheric samples where pesticide levels are generally lower than water or soil samples. The method detection limits (MDLs) required for analysis are dependent on the sample size collected and the final extract volume. Therefore the requirements for atmospheric samples with pesticides levels that are as low as 1–20 pg m^−3^ require MDLs near 1–20 pg μL^−1^ in the final extract or pg levels injected when using a standard 1 μL splitless injection. For water and soil samples a more concentrated extract can be obtained by increasing the sample size. This is not possible with atmospheric samples, as the amount of solid sorbent used to collect the gas phase fraction of pesticides and the duration of the sampling period is fixed to avoid problems with loss of pesticide during sampling due to breakthrough on sorbent materials.

When gas chromatography-mass spectrometry (GC-MS) in selected ion monitoring (SIM) mode is used for pesticide analysis the mass analyzer is most commonly a single quadrupole or ion trap (Risby, 1980; Lambropoulou and Albanis, 2001; Amendola et al. 2002; Dąbrowski et al. 2002; Jover and Bayona, 2002; Liu and Pleil, 2002; Mourabit et al. 2002; Elflein et al. 2003; Tan and Ali Mohd, 2003; Wong et al. 2003; Albero et al. 2004; Corrion et al. 2005; Ferrer et al. 2005; Gfrerer and Lankmayr, 2005; Harman-Fetcho et al. 2005; Bailey and Belzer, 2007; Beceiro-González et al. 2007). It is becoming more acceptable to use retention time (± 0.1–0.2 seconds) along with a minimum of two monitored ions, and the ratio of abundances of the two ions for added confirmation in the analysis of pesticides. The confirmation criteria generally require that this ratio lies within 20%–25% of a reference value (Elflein et al. 2003; Wong et al. 2003; Soler et al. 2006). The GC-MS ionization techniques used for pesticide analyses include electron ionization (EI), positive chemical ionization (PCI), and negative-ion chemical ionization (NCI). Mass spectral interferences particularly at low mass/charge (m/z < 100) are common in analysis of food, biological or environmental samples and more serious in EI and PCI (Bailey and Belzer, 2007). Pesticides also often do not ionize well under PCI conditions. Thus, both detection limits and specificity of the ionization mode are considered in the optimization of a GC-MS method. Selected pesticide analysis of organochlorines (OCs) or organophosphorus pesticides (OPs) by GC-MS/SIM are more commonly reported with EI (Lambropoulou and Albanis, 2001; Dąbrowski et al. 2002; Liu and Pleil, 2002; Mourabit et al. 2002; Wong et al. 2003; Elflein et al. 2003; Tan and Ali Mohd, 2003; Albero et al. 2004; Corrion et al. 2005; Ferrer et al. 2005; Gfrerer and Lankmayr, 2005; Harman-Fetcho et al. 2005; Beceiro-González et al. 2007) rather than NCI (Risby, 1980; Amendola et al. 2002; Jover and Bayona, 2002; Harman-Fetcho et al. 2005; Bailey and Belzer, 2007) as the instrumentation is less expensive, requires less skilled operators, less maintenance, and EI-MS libraries are available. These advantages of EI outweigh the decision to choose NCI for expected lower MDLs, even for OCs, which have high electro-affinity (Hunt et al. 1976; Risby, 1980; Bailey and Belzer, 2007). When analyzing atmospheric samples, pesticides have already been concentrated as much as possible. This makes the MDL of a GC-MS method the most important criteria as well as avoiding interferences through mass selection. Comparison of existing GC-MS methods are difficult as there are differences in m/z values selected even with the same ionization method, some methods use standard splitless 1 μL injections, while other methods utilize large volume injections of 10–100 μL (Risby, 1980; Bailey, 2005). In NCI, differences in m/z selection can be a function of the gas used for chemical ionization as illustrated for OPs (Amendola et al. 2002). Methane and methanol are commonly used NCI reagent gases (Amendola et al. 2002; Harman-Fetcho et al. 2005; Bailey and Belzer, 2007).

GC-tandem mass spectrometry (GC-MS/MS) is a new approach for analysis of pesticides that provides added selectivity over GC-MS SIM methods (Arrebola et al. 2003; Martinez Vidal et al. 2004; Garrido-Frenich et al. 2005). Analysis times may be reduced as the tolerance of matrix components or co-eluting pesticides increases with the more unique selected reaction monitoring (SRM) transitions. The main controversy that exists is whether tandem MS methods can provide similar sensitivity to SIM methods and whether for “real environmental samples” tandem MS methods can actually reduce the background signal and potentially minimize sample clean-up requirements for volatile matrix components. The complexity of the sample can exceed the added selectivity of the GC-MS/MS method. SRM analyses, particularly when coupled to GC systems, are still emerging and less applications than SIM analysis. The majority of available SRM analyses use electron ionization (Arrebola et al. 2003; Haib et al. 2003; González et al. 2004; Martinez Vidal et al. 2004; Garrido Frenich et al. 2005; Leandro et al. 2005; Yague et al. 2005; Ballesteros et al. 2006; Fernández Moreno et al. 2006; Garrido Frenich et al. 2006; Goncalves et al. 2006; Garrido Frenich et al. 2007; Guardia-Rubio et al. 2007).

In this paper a comprehensive comparison of GC-MS and GC-MS/MS methods using EI and NCI under the same chromatographic conditions is provided. Improvements in detection limits from those reported here would be expected with large-volume injection methods (Bailey, 2007) or for separations with only a few pesticide components and shorter retention times. This paper presents the first analysis of many of these pesticides in SRM mode particularly for NCI, along with assessment of the feasibility of the methods at levels less than 100 pg μL^−1^. For selected herbicides (triallate, trifluralin, and ethalfluralin), OCs, and OPs, NCI in either SIM or SRM mode was of interest due to its potential to increase selectivity and sensitivity when compared to the more commonly used EI-SIM analysis. Atmospheric samples are used to illustrate the issues of MS spectral interferences from matrix components and added selectivity of tandem MS or NCI methods. Selected OPs and their degradates have been analyzed by our laboratory with LC-MS/MS (Raina and Sun, 2008), however GC-MS methods are better suited for OCs and screening for a wider range of OPs.

## Experimental

### Materials

Acetone, ethyl acetate, and hexane were pesticide grade and supplied by Fisher Scientific (Ottawa, ON, Canada). Individual pesticide standards and deuterated internal standard (diazinon-d_10_ or parathion-d_10_) were supplied by Chem Service Inc. (West Chester, PA). Stock mixtures of OCs and OPs were purchased from UltraScientific (North Kingstown, RI). Standard mixtures at 1.0 μg mL^−1^ were prepared in pesticide grade hexane from solids or stock solutions and were stored at −4 °C. Suitable calibration standards were prepared by dilution of a standard mixture and internal standard (IS) with pesticide grade hexane with final concentration of internal standards, parathion-d_10_ or diazinon-d_10_ of 10 pg μL^−1^, in all standards and samples. The calibration range typically examined was 0.1–100 pg μL^−1^. All final diluted standards and samples were prepared on the day of analysis.

### Sampling site description, sample collection and preparation of air extracts

The Environment Canada sampling site at Bratt’s Lake, Saskatchewan is a field site in the prairie agricultural region where grain and oil seeds such as wheat, barley, oats, canola, and flax are predominant. Other specialty crops like lentils and peas are also grown. Weekly air samples were collected at Bratt’s Lake, Saskatchewan in 2003 using a PUF (polyurethane foam) high-volume air sampler (Tisch Environmental, Cleves, OH) with typical air volumes of 2700 m^3^. The sampling module contained the PUF/sorbent cartridge (for gas-phase) and a glass fiber filter (for particle fraction) as previously described (Bailey, 2007). The PUF/sorbent fraction consisted of 7 g of XAD-2 and 7 g TENAX-TA sandwiched between a 5.08 cm PUF (bottom) and a 2.54 cm PUF (top) was transferred after sampling into a 100 mL extraction cells and extracted with ethyl acetate using an ASE100 or ASE300 pressurized solvent extraction system (Dionex, Sunnyvale, CA). The extraction method parameters were: temperature, 100 °C; static mode time, 30 min at 1500 psi; two static cycles; 60% flush volume; purge time with nitrogen (UHP) of 300s. The total extraction volume was 150 mL. A second extraction with acetone was also tested and showed no presence of pesticides in the extracts.

After extraction, samples were concentrated to ∼5–10 mL, transferred to 15 mL vial and dried to near dryness with nitrogen or clean-room air using a solid-phase extraction (SPE) apparatus. C18 (ENVI-18, 6 mL, 1 g, Supelco Inc.) SPE tubes were conditioned with 6 mL ethyl acetate, followed by 6 mL methanol. Sample extract (0.25 mL or 0.50 mL) was loaded onto the preconditioned tubes, followed by surrogate standard (diazinon-d_10_ or parathion-d_10_, generally 0.1 mL at 1 μg/mL) and methanol such that the total volume was 1 mL. The eluted solvent collected into fraction F0 was observed to contain no pesticides of interest. The pesticides were eluted with 5 mL ethyl acetate in the next fraction (F1), and the Versiprep drying attachment (Supelco) was used to evaporate and concentrate eluted extracts from SPE (and from ASE) to near dryness. Dried extracts were redissolved with 1 mL hexane with addition of internal standard solution such that concentration of internal standard was 100 pg μL^−1^ in samples for GC-MS analysis.

### GC-MS analysis

The GC-MS/MS system consisted of an Agilent HP6890GC coupled to a tandem quadrupole mass spectrometer (GC Quattro micro) from Waters-Micromass (Milford, MA, U.S.A). The instrument had both EI and NCI capability. In SIM mode our studies found that the instrument gives comparable sensitivity to a single quadrupole instrument (Agilent 5973 Network MSD), therefore the same instrument was used for both SIM and SRM experiments.

The GC system was equipped with a split/splitless inlet with a splitless sleeve containing carbofrit (4 mm id., 6.5 × 78.5 mm, Restek, Chromatographic Specialties Inc, Brockville, ON, Canada). The injector temperature was 225 °C. A LEAP Technologies (Carrboro, NC) autosampler with a 10 μL syringe was used for injections of 1 μL at a rate of 10 μL s^−1^. The analytical column was DB-5 ms, 5% polydiphenyl-95% dimethylpolysiloxane, 30 m × 0.25 mm id and 0.25 μm film thickness (J & W Scientific, Folsom, CA) connected with a Siltek universal press-fit connector (Restek) to a short post-column (0.9 m × 0.25 mm, Siltek deactivated guard column) to allow column switching to different sources without loss of length of the analytical column. For EI the post-column was inserted to the end of the transfer line, while in NCI the post-column was inserted an additional 1–2 mm into the source to improve sensitivity. Reduced sensitivity in EI was observed when the post-column was inserted further into the ion source. The carrier gas was helium (UHP) at constant flow of 1.0 mL min^−1^. The oven temperature program had an initial temperature of 100 °C held for 1.0 minute, 15 °C/min to 250 °C held for 2.0 min, 10 °C/min to 280 °C held for 12.0 min with run time of 28.0 min. This temperature program was selected to provide adequate separation of all components with similar m/z for SIM analysis, and also for comparison of detection levels the same chromatographic conditions were used for all GC-MS and GC-MS/MS methods.

In EI SIM, the ion source temperature was 250 °C and the interface temperature was 250 °C. EI spectra were obtained at 70 eV. Collision gas was argon. For EI SRM collision cell pressure was optimized at 2.5 E-3 mbar. NCI instrumental conditions were optimized for signal intensity of test pesticide mixture of OCs and OPs prepared in hexane. In NCI the chemical ionization reagent gas was methane (99.999%) at 0.6 mL min^−1^ and argon was the collision gas for SRM at 7.5 E-3 mbar. The ion source temperature was 130 °C and the interface temperature was 250 °C for NCI. NCI spectra were obtained for SIM and SRM analysis at 60 eV with an emission current of 500 μA. Sensitivity was reduced at 70 eV. Mass calibration tuning was determined with perfluorotributylamine. Dwell times were 0.1 seconds and electron multiplier was at 650 V. The ions or SRM transitions selected and collision energies are shown in Table 1. There are 9–10 acquisition segments between 10–28 minutes in the MS methods. Each segment has approximately 16–20 masses or SRM transitions, which corresponds to 8–10 pesticides.

## Results and Discussion

### SIM and SRM selection of ions or transitions and sensitivity

The quantitative ion or SRM transition is generally the most abundant ion (or SRM transition), while the qualifier is the second most abundant ion (or SRM transition) with m/z selected above 70 to minimize the impact of interferences. For example, tokuthion in EI-SIM the most intense ions occur at m/z of 27, 29, and 43, but the selected ions were m/z of 113 and 267 ions (Table 1) to avoid interferences (Bailey and Belzer 2007). As our desired goal to was to achieve MDLs below 20 pg μL^−1^ along with confirmation at these levels, we have not considered concentrations above 100 pg μL^−1^, where in the literature the assignment of qualifier ions can be more diverse at higher concentrations than reported herein. In the literature, the qualifier ion (or SRM transition) is often an isotope peak or a higher mass product ion (particularly for chlorinated pesticides) as opposed to the second most abundant ion in the spectra. For example, chlorpyrifos, a chlorinated OP, has m/z of 97, 197, 199, and 29 as the four most abundant ions in its EI spectra, yet m/z of 314 or 316 are commonly selected (Dąbrowski et al. 2002; Mourabit et al. 2002; Liu and Pleil 2002; Tan and Mohd 2003; Harman-Fetcho et al. 2005). In our study we had to select m/z of 97, and 197 instead of m/z of 314 and 316, as these ions do not have sufficient abundance at levels < 100 pg μL^−1^. Table 2 shows that even with a higher abundance ion selected (above m/z of 70) for the qualifier, a number of pesticides do not have a reported ion ratio or have an ion ratio with relative standard deviation >25% as the qualifier ion (or SRM transition) is too weak for confirmation of identity at these concentrations. For these cases, a retention time match and an additional GC-MS method would be needed for confirmation.

In LC-MS/MS, SRM transitions are selected with the precursor ion as the protonated molecular ion to undergo collision induced dissociation (Raina and Sun 2008), however for the pesticides investigated by GC-MS methods generally the molecular ion was too weak or not visible. Table 1 shows the molecular ion is rarely one of the two most intense ions with only sulfotep, fenitrothion, parathion ethyl, sulprofos, pentachloronitrobenzene, perthane, and nitrofen observing the molecular ion as either the quantitative or qualifier ion. This high degree of fragmentation in EI leads to poor sensitivity for GC/EI-tandem MS analysis. Of all the pesticides investigated in EI-SRM, only three (sulfotep, p,p’-DDE, and o,p-DDD) had ion ratios with relative standard deviation <30% when concentrations were <100 pg μL^−1^. The use of EI-SRM was not feasible. OP pesticides with the SP(OCH_2_CH_3_)_2_ or SP(OCH_3_)_2_ structure, such as chlorpyrifos, chlorpyrfios methyl, parathion, which had significantly poorer sensitivity in EI-SRM than EI-SIM, had low abundance of the molecular ion (<10%) or of a relatively high mass precursor ion (Table 1).

The selected pre-emergent herbicides, chlorinated OPs, and OCs are electrophilic in nature and should give good sensitivity in NCI mode (Risby, 1980; Bailey and Belzer, 2007). For many of the pesticides reported in Table 1 no literature data exists in NCI-SRM mode and limited data in NCI-SIM (Amendola et al. 2002; Jover and Bayona, 2002; Haib et al. 2003; Harman-Fetcho et al. 2005; Fernández Moreno et al. 2006; Bailey and Belzer, 2007). NCI is a softer ionization process than EI so there is a tendency for less fragmentation, and in a number of cases the quantitative or qualifier ions is the molecular ion or a higher mass product ion as compared to EI (Table 1). The presence of higher mass precursor ions or the molecular ion in NCI improves the potential for lower detection limits in NCI SRM as compared to EI SRM. However for a few pesticides, such as the HCHs, a significant amount of fragmentation in the ion source is observed leading to only a small mass precursor ion (e.g. m/z = 71) available at significant abundance for collision induced dissociation (CID). Particularly when low mass chlorinated precursor ions are selected in NCI-SRM the observed ion from CID is Cl^−^ (71 > 35 for HCHs) and consequently an isotope SRM transition is often the only other available SRM transition (73 > 37 for HCHs). This trend is also observed for some higher mass product ions that have Cl atom present (aspon, pentachloronitrobenzene, heptachlor, heptachlor epoxide, chlordane, transnonachlor, endosulfan, DDT, endrin aldehyde, endrin ketone, and mirex).

### Method detection limit (MDL), limits of detection (LOD), and reproducibility, and selection of the GC-MS method

In Table 1 the most sensitive GC-MS method is identified in underline italics for each pesticide as defined by both lowest MDL and% RSD for the ion or SRM ratio used for confirmation. MDLs are calculated from the calibration curves based upon minimum concentration that shows deviation of <15% from the linear regression line. An internal standard is used in the calibration such that the response is a ratio of standard area/internal standard area. For each pesticide at least one SIM ionization method yielded MDLs in the low pg μL^−1^ for a 1 μL splitless injection with typically NCI SIM providing the best overall MDLs for the range of OCs and OPs investigated (>60% of pesticides) along with NCI-SRM which gave additional sensitivity and confirmation ability to ∼14% of pesticides. One GC-MS method could not be used for trace analysis of all pesticides. Table 2 shows that EI-SIM provides lower MDLs for only 5 of the 19 OPs examined with the remaining OPs which are halogenated having lower MDLs in combination with % RSD <25% for the confirmation ion ratio with NCI. Only a few OCs had lower MDLs in EI-SIM as compared to NCI-SIM (propachlor, alachlor, aldrin, DDT, DDE, DDD, perthane). Even for pesticides that were more sensitive in EI-SIM, NCI-SRM could be used for additional selectivity for confirmation (typically MDLs 2–25 pg μL^−1^) with the exception of OPs: sulfotep, and OCs: propachlor, alachlor, aldrin, DDT, DDE, and DDD. A few pesticides including carbofenothion, and parathion-ethyl gave optimal sensitivity with NCI-SRM making their analysis at levels <100 pg μL^−1^ feasible. The reported LODs are calculated from 3 times the signal/noise ratio obtained for a blank injection and are slightly lower or similar to the MDLs.

Reproducibility shown by%RSD in Table 2 was determined at 50 pg μL^−1^ using 10 replicate analyses. For those pesticides with good sensitivity as noted by italics in Table 2, the percentage relative standard deviation was generally <10%–20% with NCI-SIM providing the lowest RSD values. For the pre-emergent herbicides where EI-SIM, NCI-SIM, and NCI-SRM all have similar MDLs, the reproducibility is best with NCI-SIM. The correlation coefficient for the calibration curves extending from MDL to 100 pg μL^−1^ was >0.99 for all pesticides with good sensitivity.

### Criteria for confirmation: ratios of quantitative/qualifier Ion (or transition) and selectivity of GC-MS method

**Figure 1.**
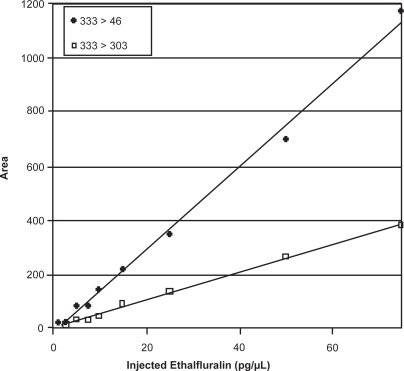
Calibration Curve for Ethalfluralin for Quantitative SRM Transition (333 > 46) and Qualifier SRM Transition (333 > 303) over Typical Calibration Range with NCI-SRM.

The confirmation ratios of quantitative/qualifier ion or SRM transition are recommended to be obtained from standards run on the day of analysis. Table 2 shows the RSD ranging in EI/SIM from 9 to ∼30%, NCI/SIM from 1.7 to ∼23%, NCI/SRM from 3.9 to ∼30% for those pesticides with better sensitivity in each mode. RSD values >25% for the confirmation ratio was either due to higher MDLs or a weak second qualifier ion (or SRM transition) such as heptachlor in EI-SIM and phorate in NCI-SRM. In the literature, the acceptance criteria of 20 or 25% RSD for the confirmation ratio has been arbitrarily set to be the same for all pesticides (Elflein et al. 2003; Wong et al. 2003; Soler et al. 2006). However, numerous pesticides observed % RSD <10% and consequently setting a criteria of 20%–25% can lead to false positives. We suggest herein that the confirmation ratio and criteria for acceptance should be based on the%RSD obtained from standards (9%–25%) with a maximum tolerance of an acceptable limit of 25%. For compounds where the confirmation ratio varies more than 25%, it is recommended that an alternative GC-MS method be used for confirmation such as both NCI-SIM and NCI-SRM. Figure 1 illustrates the consistency of the confirmation ratio where the response for the quantitative and qualifier transition for ethalfluralin in NCI-SRM is linear over the calibration range. The intensity of the qualifier SRM transition is strong until very close to the MDL. A single repeated standard injection or set of calibration standards over the linear range provides similar RSD values.

**Figure 2.**
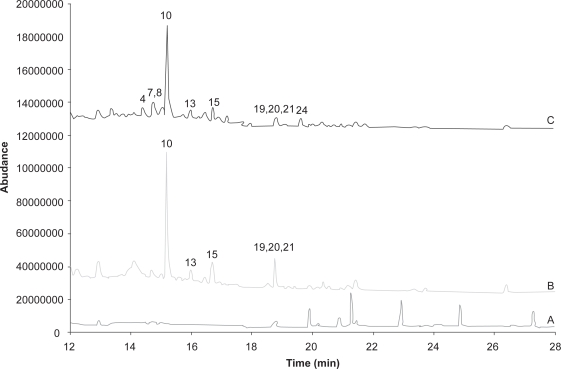
Total Ion Chromatogram for Electron Ionization in Selected ion monitoring for Quantitative Ions. A, blank air extract; B, extract from air sample from Bratt’s Lake, SK; C, extract shown in B spiked with 50 pg μL^−1^ standard mixture. Pesticides: 1, ethafluralin; 2, trifluralin; 3, phorate; 4, α-HCH; 5, diazinon-d_10_ (internal standard); 6, diazinon; 7, β-HCH; 8, γ-HCH; 9, dyfonate; 10, triallate; 11, δ-HCH; 12, dichlofenthion; 13, alachlor; 14, heptachlor; 15, chlorpyrifos; 16, DCPA; 17, trichloronate; 18, tetrachlorvinphos; 19, γ-chlordane; 20, trans-nonachlor; 21, tokuthion; 22, α-chlordane; 23, tributylphosphorotrithioite; 24, dieldrin; 25, nitrofen; 26, ethion; 27, sulprofos; 28, endosulfan sulfate; 29, endrin ketone; 30, mirex.

**Figure 3.**
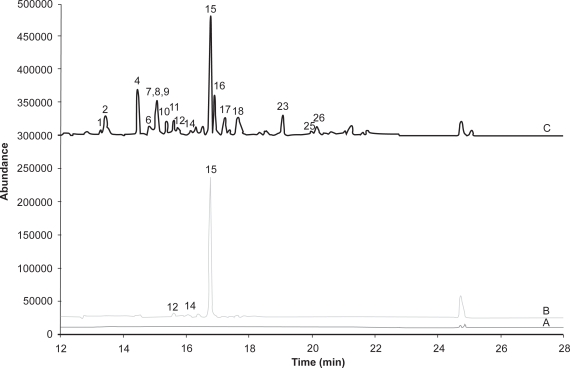
Total Ion Chromatogram for Negative-ion chemical ionization in Selected ion monitoring for Quantitative Ions. A, blank air extract; B, extract from air sample from Bratt’s Lake, SK; C, extract shown in B spiked with 50 pg μL^−1^ standard mixture. For pesticide list see Figure 2.

**Figure 4.**
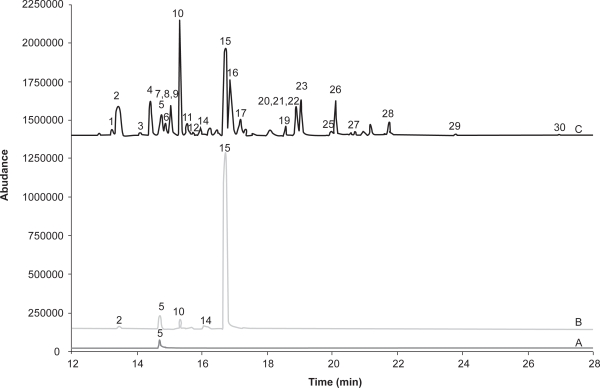
Total Selected Reaction Monitoring Chromatogram for Negative-ion chemical ionization Quantitative SRM Transitions. A, blank air extract; B, extract from air sample from Bratt’s Lake, SK; C, extract shown in B spiked with 50 pg μL^−1^ standard mixture. For pesticide list see Figure 2.

The differences in selectivity and sensitivity of GC-SIM with electron ionization (EI) and negative-ion chemical ionization (NCI) are apparent by comparison of Figures 2 and 3. The chromatogram for both the blank and air sample extract show that with GC-EI/SIM there are a number of matrix components giving additional response. These matrix components are suspected to be mainly hydrocarbon components that are not removed in the solid phase extraction cleanup procedure. Figures 3 and 4 show that NCI in either SIM or SRM mode has added selectivity with no response for the other matrix components. Figure 3 also shows that chlorpyrifos, a key OP observed in Saskatchewan air samples, has a much stronger response relative to other pesticides with GC-NCI/SIM, and is less prone to interference issues than GC-EI/SIM. GC-NCI/SRM also provides strong relative responses for chlorpyrifos, triallate, heptachlor, and trifluralin. Although not present at significant levels in the sample shown, ethalfluralin and trifluralin analyses are not impacted by interferences in GC-NCI/SIM and GC-NCI/SRM as compared to GC-EI/SIM. GC-NCI/SRM provides an additional option to GC-NCI/SIM where additional selectivity is required with typically only a small loss in sensitivity as compared to GC-NCI/SIM. Figures 2–4 also show that a good resolution of all the pesticides studied was achieved with the selected chromatographic program. Reducing the overall run time was limited in both SIM and SRM by the separation of isomers such as the HCHs and chlordane, as well as DDD, DDE, DDT. All other co-eluting peaks could be distinguished by unique ions or SRM transitions (Table 1). Although tandem-MS is often used to reduce analysis times, due to the isomers present no further reduction in retention times could be made. Chlorpyrifos, ethalfluralin, triallate, and trifluralin have been detected in all samples analyzed to date, while an additional 25 pesticides were detected with varying frequency throughout the sampling period.

**Figure 5.**
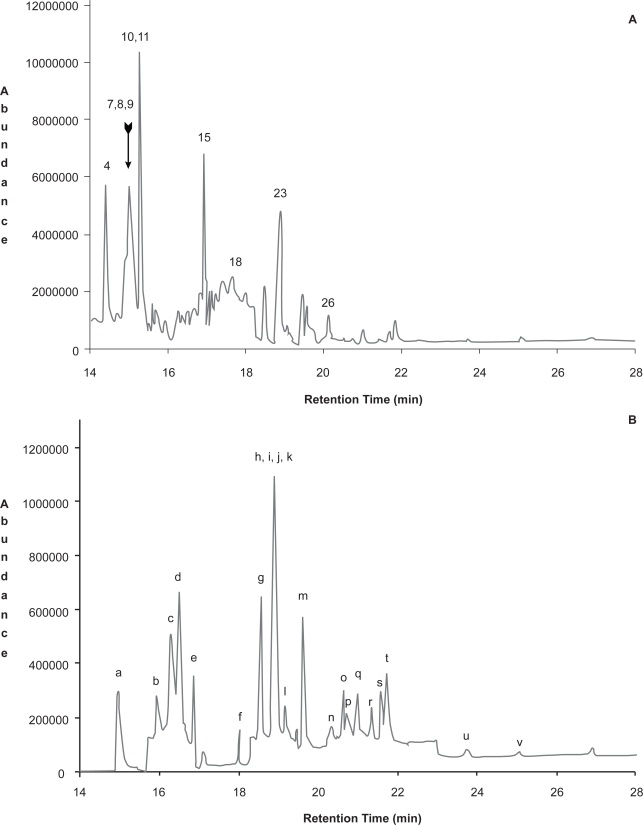
Total Selective Reaction Monitoring Chromatogram for Negative–ion Chemical ionization Selected ion monitoring for Quantitative Ions. for a 100 pg μL^−1^ standard pesticide mixture. **A:** TIC for pesticide list see Figure 2; **B:** reconstructed TIC of SIMs of only pesticides of weaker NCI-SIM response including pesticides: a, PCNB; b, chlorpyrifos methyl; c, fenchlorphos; d, malathion; e, parathion ethyl; f, heptachlor epoxide; g, gamma-chlordane; h, alpha-chlordane; i, trans-nonachlor; j, tokuthion; k, endosulfan I; l, p,p’-DDE; m, dieldrin; n, p,p’-DDD; o, endosulfan II; p, sulprofos; q, endrin aldehyde; r, p,p’-DDT; s, endosulfan sulfate; t, carbofenothion; u, endrin ketone; v, leptophos.

Figure 5 A shows the total ion chromatogram of a 100 pg μL^−1^ standard for the time period between 14 to 28 minutes, while Figure 5B shows the reconstructed chromatogram of the sum of SIMs for only those pesticides with weaker responses as noted by significantly different magnitude of the abundance. Although NCI-SIM may be the best GC-MS method of choice for a given pesticide or give a significant response, Figure 5 shows that there is a large variation in signal intensity of the SIM signal and consequently not all pesticides are observed in the overall TIC.

## Conclusion

The developed GC-EI/SIM, GC-NCI/SIM, and GC-NCI/SRM methods are suitable for the determination of pesticides in the range of MDL-100 pg μL^−1^. A single method could not be used to analyze the range of pre-emergent herbicides, OPs, and OCs investigated. However, in general GC-NCI/SIM provided the lowest MDLs along with best confirmation. GC-NCI/SRM is recommended for use where added selectivity or additional confirmation is required, and provides similar MDLs as GC-NCI/SIM. GC-EI/SRM at levels <100 pg μL^−1^ was not suitable for most pesticides. GC-EI/SIM was more prone to interference issues and although it provided similar sensitivity for the pre-emergent herbicides, it only provided lower MDLs for 5 of the 19 OPs, and 8 of the 28 OCs studied. The confirmation of a number of these pesticides in GC-EI/SIM was also limited, requiring an additional GC method for full confirmation. A three-point identification approach is recommended with area of most abundant ion (or SRM transition) used for quantitative analysis, while a second ion (or SRM transition) along with the ratio of areas obtained from the first to second ion (SRM transition) used for confirmation with sample tolerance established by the relative standard deviation of the confirmation ratio obtained from standards. When the%RSD for confirmation ratio is greater than 25%, a second GC method should be used for confirmation.

## Figures and Tables

**Table 1.** GC-MS retention time (RT) and selected ions for MS detection in SIM mode and transitions in SRM mode.

**Analyte**	**RT (min)**	**MW**	**EI-SIM (m/z)**	**EI-SRM m/z (Collision Energy (eV))**	**NCI-SIM (m/z)**	**NCI-SRM m/z (Collision Energy (eV))**
Pre-Emergent Herbicides						
Ethalfluralin	13.41	333	**276**, 316	**276** > **202 (15)**, 316 > 276 (5)	***333***, *303*	**333** > **46 (10)**, 333 > 303 (10)
Trifluralin	13.47	335	**306**, 264	**306** > **264 (5)**, 264 > 206 (5)	***335***, *305*	**335** > **305 (10)**, 335 > 46 (10)
Triallate	15.32	303	**86**, 268	**268** > **184 (15)**, 268 > 226 (10)	**160**, 161	***160*** > ***84 (15)***, *160* > *100 (10)*
Organophosphorus Pesticides (OPs)						
Sulfotep	13.71	322	***322***, *202*	**322** > **202 (10)**, 322 > 146 (25)		
Phorate	14.19	260	***121***, *75*	**260** > **75 (5)**, 263 > 231 (5)	**222**, 224	**185** > **111 (15)**, 185 > 157 (10)
Diazinon –d_10_ (IS)	14.70	314	**314**	**314** > **185 (15)**	**314**	**179** > **95 (20)**
Diazinon	14.75	304	***137***, *179*	**304** > **179 (5)**, 304 > 137(30)	**169**, 171	**169** > **95 (20)**, 169 > 141 (10)
Dyfonate	15.09^a^	246	**109**, 137	**246** > **137 (5)**, 246 > 109 (15)	***169***, *109*	**169** > **107 (20)**, 169 > 141 (5)
Dichlofenthion	15.73	314	**223**, 97	**314** > **223 (25)**, 319 > 81 (25)	***278***, *250*	
Chlorpyrifos methyl	15.93	321	**286**, 125	**321** > **268 (5)**, 321 > 208 (20)	***214***, *212*	**141** > **126 (15)**, 141 > 96 (20)
Fenchlorphos	16.26	320	**125**, 287	**320** > **285 (5)**, 320 > 204 (30)	***211***, *213*	**141** > **126 (15)**, 211 > 35 (20)
Aspon	16.38	378	***211***, *253*	**378** > **210 (10)**, 378 > 115 (30)		**211** > **35 (20)**
Fenitrothion	16.59^a^	277	**277**, 125	**277** > **260 (5)**, 277 > 109 (20)	***277***, *168*	
Malathion	16.50^a^	330	**173**, 125		***172***, *157*	**157** > **142 (15)**, 172 > 84 (5)
Chlorpyrifos ethyl	16.77	349	**97**, 197	**349** > **208 (10)**, 349 > 40 (5)	***313***, *315*	**169** > **95 (15)**, 313 > 189 (10)
Parathion ethyl	16.97	291	**97**, 291	**291** > **109 (10)**, 291 > 137 (10)	**291**, 292	***291*** > ***154 (5)***, *291* > *169 (10)*
Trichloronate	17.21	332	**109**, 297		**213**, 296	***296*** > ***108 (10)***, *296* > *79 (20)*
Tokuthion	18.90	344	**113**, 267	**344** > **328 (10)**, 344 > 73 (20)	**237**, 301	***269*** > ***161 (10)***, *237* > *79 (25)*
Tributylphosphorotrithioite	19.06	314	**169**, 57	**314** > **115 (20)**, 314 > 113 (20)	**257**	***257*** > ***79 (15)***, *257* > *89 (20)*
Ethion	20.12	384	**231**, 97	**384** > **231 (5)**, 384 > 203 (15)	***185***, *187*	**185** > **111 (15)**, 185 > 157 (15)
Sulprofos	20.75	322	***140***, *322*	**322** > **156 (10)**, 322 > 97 (20)	**279**, 247	**279** > **139 (20)**, 279 > 124 (35)
Carbofenothion	21.73^a^	342	**157**, 342	**342** > **157 (10)**, 342 > 143 (15)	**185**, 145	***185*** > ***111 (20)***, *185* > *79 (35)*
Leptophos	25.04	410	**171**, 377		***241***, *79*	**241** > **81 (35)**, 257 > 81 (35)
Organochlorine Pesticides (OCs)						
Propachlor	13.41	211	***120***, *176*	**176** > **120 (10)**, 176 > 92 (15)		
a-HCH	14.43	288	**181**, 111	**181** > **145 (15)**, 181 > 109 (25)	***71***, *73*	**71** > **35 (5)**, 73 > 37 (10)
b-HCH	14.91	288	**181**, 111	**181** > **145 (15)**, 181 > 109 (25)	***71***, *73*	**71** > **35 (5**), 73 > 37 (10)
Pentachloronitrobenzene	14.96^a^	293	**237**, 295	**237** > **119 (15)**, 237 > 146 (25)	***265***, *249*	**265** > **35 (35)**, 231 > 35 (25)
g-HCH	14.99	288	**181**, 111	**181** > **145 (15)**, 181 > 109 (25)	***71***, *73*	**71** > **35 (5)**, 73 > 37 (10)
d-HCH	15.56	288	**111**, 181	**181** > **145 (15)**, 181 > 109 (25)	***71***, *73*	**71** > **35 (5)**, 73 > 37 (10) (25)
Alachlor	15.98	269	***160***, *188*	**188** > **160 (10)**, 160 > 130 (25)		
Heptachlor	16.43	370	**100**, 274	**100** > **65 (10)**, 272 > 237 (10)	***266***, *232*	**266** > **35 (30)**, 300 > 35 (25)
DCPA	16.90	330	**301**, 332	**332** > **301 (5)**, 301 > 273 (15)	***332***, *330*	**330** > **35 (10**), 332 > 302 (5)
Aldrin	17.21	362	***79***, *263*	**263** > **193 (35)**, 263 > 228 (15)	**330**	
Heptachlor epoxide	18.01	386	**81**, 353	**353** > **263 (15)**, 353 > 317 (5)	***282***, *318*	**71** > **35 (5)**, 282 > 35 (20)
o,p’-DDE	18.40	316	***246***, *318*	**246** > **176 (15)**, 316 > 246 (25)	**316**, *248*	
g-Chlordane	18.54	406	**375**, 373	**375** > **266 (25)**, 375 > 303 (10)	***266***, *71*	**71** > **35 (5)**, 266 > 35 (15)
a-Chlordane	18.86	406	**373**, 375	**375** > **266 (25)**, 375 > 303 (10)	***266***, *232*	**71** > **35 (5)**, 266 > 35 (15)
Trans-nonachlor	18.90	440	**409**, 407	**409** > **302 (25)**, 409 > 265 (20)	***444***, *442*	**446** > **35 (5)**; 300 > 35 (25)
Endosulfan I	18.94	404	**241**, 195	**195** > **160 (5)**, 339 > 159 (15)	***372***, *404*	**242** > **35 (15)**, 406 > 35 (10)
p,p’-DDE	19.18	316	***316***, *318*	**316** > **246 (15)**, 246 > 176 (25)	**318**, *316*	
o,p’-DDD	19.43	306	***235***, *165*	**235** > **165 (25)**, 235 > 200 (5)	**71**, 248	
Dieldrin	19.65	378	**79**, 263	**263** > **193 (35)**, 263 > 228 (15)	***346***, *380*	
Perthane	19.72	223	***223***, *224*	**223** > **194 (10)**, 224 > 195 (10)	**217**	**217** > **35 (20)**
Nitrofen	20.06^a^	283	**283**, 285	**283** > **162 (20)**, 283 > 253 (10)	***283***, *138*	**283** > **138 (1**0**)**, 283 > 35 (35)
p,p’-DDD	20.39	306	***235***, *165*	**235** > **165 (25)**, 235 > 200 (5)	**71**, 248	**71** > **35 (5)**, 73 > 37 (5)
Endosulfan II	20.56	404	**241**, 195	**195** > **159 (10)**, 339 > 125 (35)	***372***, *406*	**242** > **35 (15)**, 406 > 35 (10)
Endrin aldehyde	21.00	378	**67**, 245	**345** > **281 (10)**, 345 > 245 (10)	**272**, 270	**272** > **35 (20)**, 272 > 243 (15)
p,p’-DDT	21.58	352	**235**, 165	**235** > **165 (25)**, 235 > 199 (15)	**71**, 73	**71** > **35 (5)**
Endosulfan sulfate	21.70^a^	420	**387**, 272	**272** > **237 (25)**, 387 > 253 (10)	**386**, *352*	**386** > **97 (10)**, 352 > 97 (15)
Endrin ketone^a^	23.71	378	**317**, 67	**317** > **281 (10)**, 317 > 245 (20)	**308**, *272*	**308** > **35 (35)**, 308 > 272 (15)
Mirex^a^	26.88	540	**272**, 274	**272** > **237 (10)**, 272 > 141 (30)	**402**, *368*	**368** > **35 (20)**, 404 > 35 (15)

First ion or SRM transition (in bold): quantification, second ion or SRM: qualifier. Best GC-MS method in underline italics. Retention time listed determined from EI-SIM except for ^a^from NCI-SIM and ^b^from NCI-SRM.

**Table 2.** Detection Limits, Reproducibility, and Ion or SRM Response Ratio for MS detection.

**Analyte**	**NCI-SIM**				**NCI-SRM**				**EI-SIM**			
	**LOD**	**MDL**	**% RSD**	**Ion ratio** ±**% RSD**	**LOD**	**MDL**	**% RSD**	**SRM ratio** ±**% RSD**	**LOD**	**MDL**	**% RSD**	**Ion ratio** ±**% RSD**
Pre-Emergent Herbicides												
Ethalfluralin	*2.5*	*5.0*	*13.0*	*5.12* ± *5.0*	3.3	5.0	14.2	2.57 ± 13.9	10.2	15.0	18.1	1.76 ± 2.1
Trifluralin	*1.5*	*2.5*	*9.4*	3*.33* ± *1.7*	3.4	5.0	11.5	0.90 ± 3.9	2.7	5.0	15.6	0.97 ± 9.1
Triallate	1.5	2.5	6.4	8.66 ± 14.8	*2.1*	*5.0*	*7.3*	*1.13* ± *4.3*	1.1	5.0	14.8	9.89 ± 13.7
Organophosphorus Pesticides (OPs)												
Sulfotep									*1.8*	*5.0*	*12.7*	*1.42* ± *9.00*
Phorate	7.8	15.0	11.4		9.8	15.0	14.8	5.10 ± 45.0	*10.0*	*15.0*	*14.6*	*0.17* ± *10.*2
Diazinon	3.9	5.0	*4.3*	*20.1* ± *26.6*	8.4	10.0	13.8	2.27 ± 20.5	*0.7*	*2.0*	*16.8*	*2.62* ± *9.7*
Dyfonate	*1.0*	*2.5*	*6.4*	*3.71* ± *2.0*	5.2	7.5	6.6	1.77 ± 8.6				
Dichlofenthion	*5.0*	*7.5*	*11.9*	*4.86* ± *13.2*					19.0	50.0	46.9	
Chlorpyrifos methyl	*7.6*	*10.0*	*10.9*	*1.00* ± *3.6*	11.7	15.0	13.8	3.07 ± 24.0	10.8	25.0	18.5	0.30 ± 22.2
Fenchlorphos	*7.5*	*10.0*	*11.1*	*1.01* ± *1.7*	27.2	50.0	18.8		12.5	50.0	28.9	3.07 ± 30.2
Aspon					21.9	25.0	25.0		13.1	15.0	20.5	1.05 ± 29.3
Fenitrothion	*36.6*	*75.0*	*15.6*	*1.1* ± *19.2*								
Malathion	*9.5*	*15.0*	*6.3*	*4.82* ± *8.2*	27.6	50.0	20.2					
Chlorpyrifos ethyl	*5.1*	*7.5*	*13.0*	*1.39* ± *3.7*	4.5	5.0	11.7	3.64 ± 22.1	15.5	25.0	13.8	6.45 ± 32.0
Parathion ethyl	32.0	50.0	11.1	6.10 ± 21.0	*9.7*	*15.0*	*14.7*	*3.25* ± *35.2*	18.7	50.0	21.2	
Trichloronate	2.6	10.0	6.6	0.30 ± 15.2	*7.4*	*7.5*	*11.7*	*2.41* ± *18.5*	6.2	7.5	15.0	8.96 ± 26.4
Tokuthion	7.3	7.5	6.0	1.15 ± 31.6	*4.7*	*7.5*	*11.4*	*4.13* ± *20.9*	22.0	50.0	28.1	
Tributylphosphorotrithioite	2.0	2.5	7.4		*0.5*	*5.0*	*18.8*	*1.17* ± *12.8*	8.5	15.0	23.4	0.13 ± 23.2
Ethion	*3.8*	*5.0*	*5.2*	*11.5* ± *10.2*	4.1	10.0	10.6	3.73 ± 22.1	5.6	15.0	2.2	0.17 ± 17.5
Sulprofos	8.2	15.0	11.0	4.74 ± 37.9	9.6	10.0	10.4	1.54 ± 30.5	*2.6*	*10.0*	*16.9*	*3.68* ± *11.9*
Carbofenothion	14.4	15.0	25.3		*6.8*	*15.0*	*13.1*	*3.54* ± *27.5*				
Leptophos	*6.1*	*7.5*	*21.1*	*10.1* ± *19.4*					5.4	25.0	24.8	
Organochlorine Pesticides (OCs)												
Propachlor									*1.8*	*5.0*	*17.4*	*6.76* ± *23.6*
a-HCH	*1.9*	*2.5*	*13.4*	*1.54* ± *0.60*	2.0	2.5	15.9	3.23 ± 7.5	3.2	7.5	12.8	0.64 ± 9.8
b-HCH	*6.2*	*7.5*	*8.8*	*1.49* ± *2.0*	9.2	15.0	13.5	3.28 ± 10.1	15.7	15.0	32.3	
Pentachloronitrobenzene	*4.6*	*7.5*	*11.3*	*1.30* ± *4.9*	15.4	25.0	13.5	3.47 ± 49.1				
g-HCH	*1.2*	*2.5*	*10.6*	*1.58* ± *2.4*	3.4	5.0	16.2	3.39 ± 7.0	3.5	10.0	14.0	0.70 ± 13.8
d-HCH	*5.0*	*7.5*	*9.3*	*1.61* ± *4.0*	7.2	7.5	26.6	3.09 ± 33.9	18.9	50.0	46.4	
Alachlor									*2.0*	*2.5*	*12.7*	*2.25* ± *13.9*
Heptachlor	*8.1*	*10.0*	*4.9*	*1.34* ± *13.1*	29.3	25.0	14.1	5.63 ± 46.8	2.8	5.0	17.5	72.5 ± 58.7
DCPA	*0.5*	*1.0*	*5.3*	*1.21* ± *1.4*	3.0	5.0	10.4	7.99 ± 12.5	5.9	7.5	28.2	2.66 ± 24.8
Aldrin									*12.6*	*15.0*	*20.1*	*9.16* ± *27.5*
Heptachlor epoxide	*8.2*	*10.0*	*7.5*	*0.67* ± *9.6*	6.4	7.5	16.0	18.0 ± 27.4	7.0	15.0	1.1	40.7 ± 23.8
o,p’-DDE	25.7	50.0	16.0	0.65 ± 52.5					*2.3*	*5.0*	*9.6*	*8.75* ± *16.3*
g-Chlordane	*3.5*	*5.0*	*4.6*	*0.38* ± *9.6*	4.8	5.0	13.8	4.88 ± 11.3	18.4	25.0	20.1	0.91 ± 31.0
a-Chlordane	*6.9*	*10.0*	*12.1*	*1.05* ± *5.9*	2.8	5.0	15.3	24.0 ± 12.7	27.1	50.0	32.1	1.53 ± 63.8
Trans-nonachlor	*4.4*	*5.0*	*6.0*	*1.19* ± *2.1*	11.9	15.0	14.1	2.72 ± 11.5	22.2	15.0	22.9	1.06 ± 21.1
Endosulfan I	*3.6*	*5.0*	*7.3*	*1.66* ± *6.2*	12.6	15.0	16.3	5.57 ± 12.8	9.7	15.0	52.1	0.53 ± 60.7
p,p’-DDE	8.5	15.0	10.3						*4.8*	*5.0*	*12.0*	*0.80* ± *12.2*
o,p’-DDD	20.3	25.0	27.7						*12.9*	*25.0*	*7.1*	*0.53* ± *16.2*
Dieldrin	*3.7*	*7.5*	*5.8*	*3.21* ± *6.7*					2.2	5.0	14.1	51.2 ± 27.3
Perthane									*1.3*	*2.5*	*12.0*	*4.85* ± *10.8*
Nitrofen	*6.28*	*25.00*	*11.1*	*1.64* ± *8.7*	6.1	15.0	19.4	6.62 ± 37.7				
p,p’-DDD	33.6	75.0	14.2						*4.9*	*7.5*	*19.1*	*0.59* ± *18.6*
endosulfan II	*4.5*	7.5	*6.5*	*0.56* ± *9.2*	32.0	50.0	16.8	6.61 ± 22.2	35.3	50.0	36.1	
Endrin aldehyde	*10.6*	*25.0*	*6.6*	*1.03* ± *4.4*	6.8	25.0	16.5	0.52 ± 49.0	9.63	25.0	15.1	28.7 ± 28.9
p,p’-DDT	*13.2*	*15.0*	*5.6*	*1.54* ± *5.4*					22.7	25.0	22.2	
Endosulfan sulfate	*6.0*	*7.5*	*7.1*	*6.56* ± *9.7*6	3.5	5.0	11.0	9.84 ± 25.6				
Endrin ketone	*23.2*	25.0	13.6	2*.92* ± *23.*2	59.9	100.0	27.4					
Mirex	*7.0*	*25.0*	*13.6*	*0.78* ± *14.7*	52.7	100.0	25.4					

Ion or SRM ratio (quantitative/qualifier) determined from areas of a 50 pg μL^−^1 standard (N = 10). Best GC-MS method in underline italics.
